# 30 Dynapenia and risk factors in Immune-Mediated Rheumatic Diseases: A Systematic Review

**DOI:** 10.1093/eurpub/ckae114.163

**Published:** 2024-09-26

**Authors:** Rafaela Espírito Santo, Geiziane Melo, Cesar Agostinis-Sobrinho

**Affiliations:** Klaipeda University, Klaipeda, Lithuania; Klaipeda University, Klaipeda, Lithuania; Klaipeda University, Klaipeda, Lithuania

## Abstract

Dynapenia, the age-related decline in muscle strength, poses significant challenges to the quality of life, autonomy, and health of individuals, becoming a major public health concern. Immune-Mediated Rheumatic Diseases (IMRDs) patients often experience dynapenia, regardless of age. Therefore, our aims were to assess the association between changes in muscle strength and changes in clinical features in IMRDs. Additionally, to evaluate risk factors for adverse health outcomes in IMRDs. A systematic review of longitudinal studies published in English was conducted using PubMed, Embase, Web of Science and Scopus to November 2023. Search strategies were based on pre-defined keywords and medical subject headings. The methodological quality of included studies was assessed using the Newcastle-Ottawa Scale. Of 11.692 potential studies (5138 duplicate publication) screened for inclusion in the study, twenty-one were included. Of these 21 studies included, one study was with systematic sclerosis (Ss) patients, two studies were with systemic erythematosus lupus (SLE) patients and eighteen studies were with rheumatoid arthritis (RA) patients. The decrease in muscle strength was associated with worsening clinical features over time in both SLE and RA patients. Lastly, low muscle strength was linked to multiple falls and reduced 5-year survival in RA patients. Therefore, changes in muscle strength are associated with changes in clinical features over time, and low muscle strength is linked to adverse health outcomes. The study emphasizes the importance of targeted exercise interventions aimed at improving muscle strength to mitigate adverse health outcomes and enhance clinical features in IMRDs patients.

